# Human Papillomavirus Type 16 Disables the Increased Natural Killer Cells in Early Lesions of the Cervix

**DOI:** 10.1155/2019/9182979

**Published:** 2019-04-28

**Authors:** Jiexin Zhang, Shanshan Jin, Xiao Li, Lenan Liu, Lei Xi, Fang Wang, Shichang Zhang

**Affiliations:** ^1^Department of Laboratory Medicine, The First Affiliated Hospital of Nanjing Medical University, 210029 Nanjing, China; ^2^Department of Oncology, The First Affiliated Hospital of Nanjing Medical University, 210029 Nanjing, China; ^3^Department of Pathology, The First Affiliated Hospital of Nanjing Medical University, 210029 Nanjing, China; ^4^Department of Obstetrics, The First Affiliated Hospital of Nanjing Medical University, 210029 Nanjing, China

## Abstract

The mechanism for pathogenesis of human papillomavirus (HPV) in the cervix has been investigated intensively. However, detailed differences in the distribution and function of innate immune cells between high-risk HPV types, especially during the chronic inflammation phase, have not been described fully. In this study, histologic pathology results of 245 women with HPV type 16 only (HPV16^+^) or type 18 only (HPV18^+^) were analyzed retrospectively from January 2015 to November 2016. More severe lesions of the cervix were observed in HPV16^+^ women compared with those in HPV18^+^ women. In total, 212 cervical brush specimens were collected from women suffering from chronic inflammation, HPV16^+^, or HPV18^+^ from December 2016 to December 2018. Flow cytometry analysis showed that abundant NK cells along with aberrant Treg cells were found in the HPV16-infected cervix. Quantitative real-time PCR demonstrated that higher expression levels of IFN-*γ* but muted IL-2 and KLRG-1 expression was detected in the cervix of patients with HPV16^+^ compared to HPV18^+^, which were further confirmed using 20 paraffin sections of cervical conization tissue. The ex vivo cytotoxicity experiment showed that the cytotoxicity of NK cells was significantly decreased in the cervix of HPV16^+^ patients compared with that of HPV18^+^ patients. Collectively, our results suggested that HPV16 disables the increased NK cells in the early lesion of the cervix, indicating that the local immune system of the cervix is hyporesponsive to HPV16 infection and this may explain its bias for malignant transformation.

## 1. Introduction

Based on the most recent data, the immune system of most people infected with HPV needs at least three years in order to eliminate the virus. Apart from an inadequate humoral immune response, suppression of immune cells in the local cervical environment is another major reason for these statistics [1]. HPV invades vaginal squamous epithelium as well as undifferentiated basal cells within the cervical canal epithelium transitional zone [2]. So far, more than 100 HPV types have been identified, among which 15 types (such as HPV types 16, 18, 31, and 45) are considered high-risk types which are more likely to be carcinogenic [3]. A recent worldwide comprehensive survey indicated that up to half of cervical cases is caused by HPV type 16 (HPV16), and HPV16 and HPV type 18 (HPV18) together lead to up to 80% [4, 5].

Studies have provided solid evidence on the molecular mechanisms by which HPV infiltrates local immune cells. NK cells are important biological barriers which are resident in the cervix. They can identify virus-infected or virus-transformed cells rapidly through pathways that do not require presensitization and kill them. It has been reported that NK cells emerge at an early stage in HPV-infected lesions [6]. However, HPV can force activate NK cells to lose their membrane receptors thus leading to their malfunction and this can lead to carcinogenesis [7]. A reversal of the CD4^+^/CD8^+^ ratio and a marker increase in the number of Treg cells, which are the representative immunosuppressive cells, have also been shown in the crosstalk between cervical cancer cells and the virus [8, 9]. Nevertheless, there is no report so far focusing on the profiles of these infiltrated immune cells in tissues infected with different HPV types. The purpose of this study was to compare the distribution and functional status of innate NK cells in tissue infected with two high-risk HPV types (16 and 18) and to discuss the possible mechanisms of HPV-original disease from a different perspective.

## 2. Materials and Methods

### 2.1. Patient Characteristics

Eligible patients were married women aged between 25 and 65 without histologically or cytologically confirmed other diseases originating from the reproductive system (including the vagina, uterus, and ovary) but with cervical chronic inflammation only. This research was authorized by the Ethical Committee of the First Affiliated Hospital of Nanjing Medical University (Nanjing, China).

### 2.2. Sample Collection

We used a speculum to expose the cervical canal, and the coherent mass of tissue was cleared with a cotton swab. A cervical brush was inserted into the cervical canal to approximately 1-1.5 cm and was gently rotated in the same direction for 5 times. Then, the cervical brush was slowly extracted, and the exfoliated cells were preserved in a Roche® sample collection tube for type detection or in 1640 cell culture medium containing 10% FBS. In total, 212 cervical brush specimens were collected for flow cytometry analysis, ex vivo cytotoxicity detection, and quantitative real-time PCR from December 2016 to December 2018.

### 2.3. HPV DNA Detection and Type Identification

HPV DNA detection and type identification were performed using a cobas® x480 nuclear acid extraction system and a cobas® z480 analyzer via a cobas® 4800 system liquid cytology preparation kit according to the manufacturer's instructions (Roche, USA). Specimens with HPV16 or HPV18 were, respectively, preserved as the HPV16^+^ group or HPV18^+^ group. Specimens derived from women with chronic inflammation and HPV negative were used as controls.

### 2.4. Fluorescence-Activated Cell Sorting (FACS)

Preserved samples were washed three times with PBS before passing through a 40 *μ*m strainer filter twice (STEMCELL Technologies, Canada). Remaining cells were conjugated to CD3, CD4, CD56/CD16 (Beckman Coulter), and either CD16 or CD25 antibodies in combination (Biolegend, USA) and subjected to flow cytometry (CD56/16 with Beckman, others with BD FACSCalibur, USA). The FACS results show the percentages of each indicated immune cell type.

### 2.5. RNA Extraction and Quantitative Real-Time PCR

The total RNA of cervical brush specimens was extracted using a TRIzol Reagent (Grand Island, USA) according to the manufacturer's instructions. Twenty paraffin sections of cervical conization tissue were subjected to quantitative real-time PCR. The unstained sections were deparaffinized using a standard xylene and ethanol procedure. RNA was extracted using RNeasy FFPE Kits (QIAGEN) as per the manufacturer's instructions.

The reverse transcription reaction from 1 *μ*g of RNA template was carried out using a First Strand cDNA Synthesis Kit (TOYOBO, Japan). Quantitative real-time PCR was performed using SYBR Green Real-time PCR Master Mix (TOYOBO, Japan) and detected by the LightCycler 480 II real-time PCR system (Roche, USA). The expression level was analyzed and normalized to *β*-actin in the cDNA samples. The fold change of gene expression was calculated using the 2^-ΔΔCT^ method. The primer sequences are listed in Table 1.

### 2.6. *Ex Vivo* Immune Cell Cytotoxicity Experiments

The cervical cancer cell line HeLa cells were labeled with CFSE (0.5 *μ*M, Invitrogen). CD56^+^CD16^+^ NK cells were isolated from cervical brush specimens of women infected with HPV16 or HPV18 via FACS (BD FACSAria II, USA). NK cells were cocultured with CFSE-HeLa in designated ratios in 24-well plates and incubated at a 37°C incubator for 4-6 hours. Cells were harvested, stained with 7-AAD (BD Pharmingen, USA), and subjected to flow cytometry (BD FACSCalibur, USA). Data were analyzed using the FlowJo 6.0 software (Tree Star).

### 2.7. Statistical Analysis

Statistical analyses were performed using PRISM 7.01 (GraphPad Software Inc., San Diego, CA). Data are presented as the means ± SD. All data were analyzed using Student's *t*-test, where *p* < 0.05 was considered statistically significant. The Bonferroni correction was used for multiple comparisons.

## 3. Results

### 3.1. Overall Histologic Pathology Profiles of HPV Type-Infected Women

We retrospectively analyzed the histologic pathology results of 245 HPV16- or HPV18-infected women from January 2015 to November 2016. As shown in Table 2, more severe lesions (≥CIN 2) were statistically presented in women with HPV16 infection when compared with HPV18^+^ women.

### 3.2. NK Cell Infiltration in a HPV16-Infected Cervix

**Figure 1:**
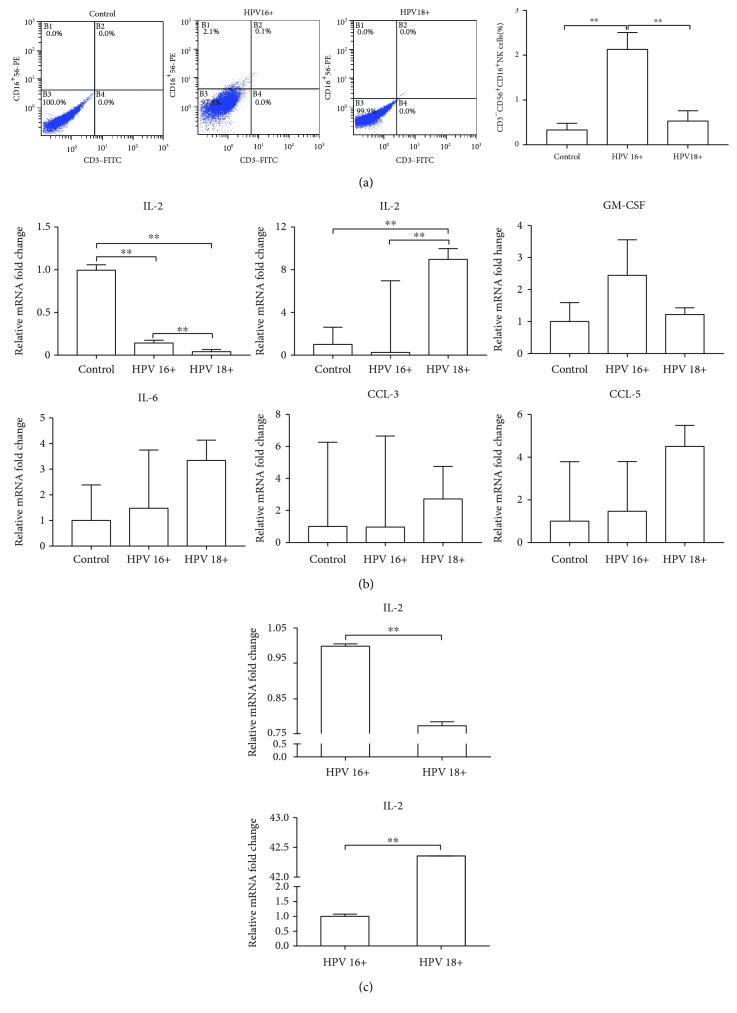
The differences of NK cells between HPV16- and HPV18-infected cervixes. (a) Distribution of CD56^+^CD16^+^ NK cells was analyzed in the cervical brush specimens by FACS. (b) Several soluble cytokines that represent NK cell involvement in the immune status were investigated in the cervical brush specimens by quantitative real-time PCR. (c) IFN-*γ* and IL-2 expressions were measured in the cervical conization tissue by quantitative real-time PCR. ^∗^*P* < 0.05; ^∗∗^*P* < 0.01.

Both HPV16 and HPV18 are categorized into high-risk HPV types that have a strong probability to cause cervical invasive carcinoma. We wondered whether the distribution patterns of CD56^+^CD16^+^ representing NK cells were similar between these two types of virus. In total, 48 cervical brush specimens including 16 chronic inflammation controls, 16 HPV16^+^ cases, and 16 HPV18^+^ cases were collected. As shown in Figure 1(a), compared to chronic inflammation control, CD56^+^CD16^+^ NK cells were upregulated in both HPV16^+^ and HPV18^+^ cervical surfaces. Interestingly, the ratio was even more abundant in the HPV16^+^ group than that in the HPV18^+^ group (CD56^+^CD16^+^ NK cells 2.13 ± 0.38% vs. 0.53 ± 0.23%; *p* < 0.01). Cell smear inspection also showed a relatively high density of fried egg-like NK cells in the HPV16^+^ group.

The second batch of cervical brush specimens consisted of 25 chronic inflammation controls, 22 HPV16^+^ cases, and 15 HPV18^+^ cases that were collected to evaluate six soluble cytokines that represented the NK cell-involved immune status in the cervical microenvironment. As shown in Figure 1(b), IFN-*γ* transcription level was relatively higher in the HPV16^+^ group than that in the HPV18^+^ group, which was consistent with elevated numbers of local NK cells. But despite the viral types, HPV infection induced a decreasing trend of IFN-*γ* transcription at the cervical surface. IL-2 is an important cytokine in microbial infection and bridges the body's innate and adaptive immune systems. We found that IL-2 levels were significantly lower in the HPV16^+^ group indicating that the local immune response failed to be further stimulated by virus infection. On the contrary, HPV18 infection could. Other tested cytokines such as IL-4, GM-CSF, CCL-3, and CCL-5 showed no significant difference between infections with the two HPV types. To verify these changes in cervical brush specimens, we again examined women who were pathologically diagnosed with cervical chronic inflammation and collected paraffin sections of cervical conization tissue from those infected with HPV16 (10 cases) or HPV18 (10 cases). As shown in Figure 1(c), the trends of the IFN-*γ* and IL-2 expression difference between the two groups were identical. Taken together, the HPV16^+^ cervix is characterized by the increased population of NK cell proportion and a distinguished cervical microenvironment.

### 3.3. NK Cells Exert a Discorded Effect in a HPV16^+^ Cervix

**Figure 2:**
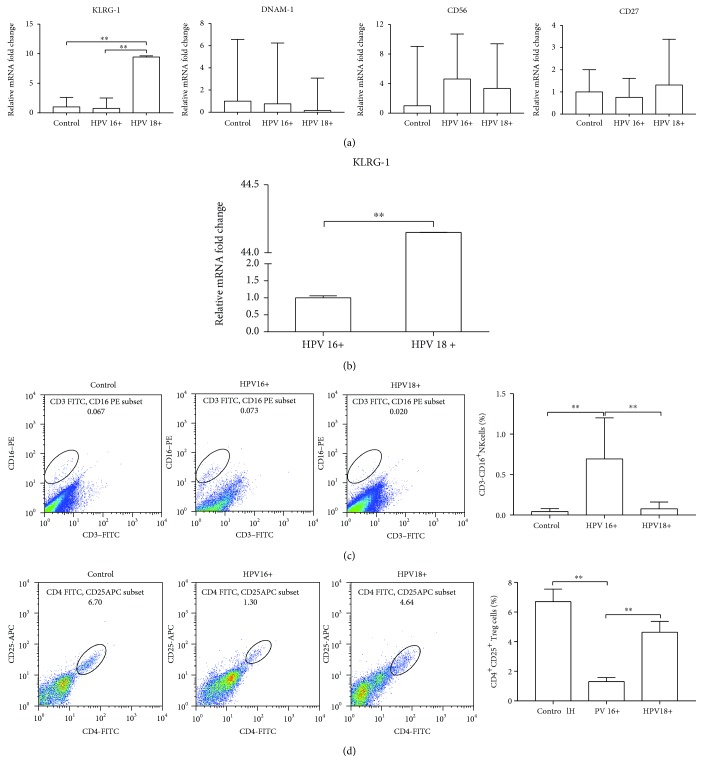
The different effects of NK cells between HPV16- and HPV18-infected cervixes. (a) Four typical cell membrane markers of NK cells were evaluated in the cervical brush specimens by quantitative real-time PCR. (b) KLRG-1 expression was measured in the cervical conization tissue by quantitative real-time PCR. (c) Distribution of CD16^+^ NK cells was analyzed in the cervical brush specimens by FACS. (d) Distribution of CD4^+^CD25^+^ Treg cells was analyzed in the cervical brush specimens by FACS. ^∗^*P* < 0.05; ^∗∗^*P* < 0.01.

We also tested four typical cell membrane markers of NK cells in the second batch of specimens. KLRG-1 is upregulated in cells designated as vibrant NK cells possessing a cytotoxicity effect. But its expression was dull in the HPV16^+^ group (Figure 2(a)), indicating that it fails to respond when the virus is mature and proceed to the next biological cycle at the cervical surface. This result was also confirmed in the paraffin sections (Figure 2(b)). CD56 transcription levels of both HPV groups were similar or slightly higher than control (Figure 2(a)). This does not agree with the result from flow cytometry studies (Figure 1(a)). Considering that the combined CD56/CD16 antibody used in Figure 1 was designed for clinical use only, we chose another different commercial CD16 antibody. The flow cytometry analysis of the third batch of cervical brush specimens (16 cases in each group) showed that CD16^+^ cells were markedly increased in HPV16^+^ group (HPV16^+^0.693 ± 0.507% vs. control 0.043 ± 0.037% and HPV18^+^0.077 ± 0.083%; Figure 2(c)).

**Figure 3:**
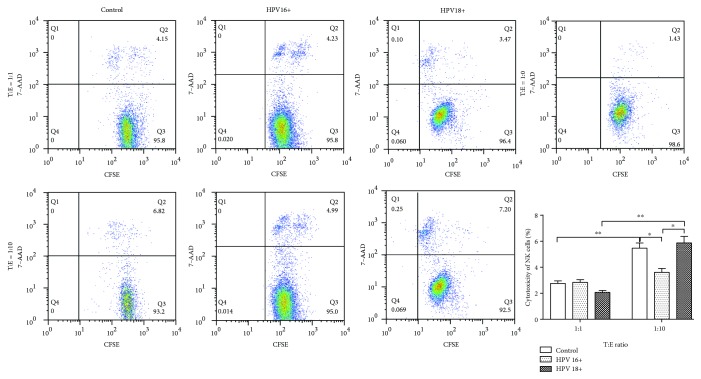
The cytotoxicity of NK cells derived from HPV16^+^ or HPV18^+^ women using HeLa cells labeled with CFSE. CD56^+^CD16^+^ NK cells were isolated from cervical brush specimens of women infected with either HPV16 or HPV18, respectively, via FACS. NK cells were cocultured with CFSE-HeLa in designated ratios in 24-well plates and incubated at a 37°C incubator for 4-6 hours. Cells were harvested and stained with 7-AAD. CFSE^+^7-AAD^+^ cells were examined by FACS. ^∗^*P* < 0.05; ^∗∗^*P* < 0.01.

Treg cells play an important role in “self-checking” the regulatory immune response, and there is a trade-off between them and NK cells which is believed to have a great contribution towards HPV-induced carcinogenesis [10]. To investigate whether increased infiltration of NK cells implied Treg recession, 48 cervical brush specimens were analyzed by flow cytometry. As expected, the CD4^+^CD25^+^ Treg cell ratio in the HPV16^+^ group was the lowest among the three groups (HPV16^+^1.30 ± 0.28% vs. control 6.70 ± 0.85% and HPV18^+^4.64 ± 0.73%; Figure 2(d)). The cytotoxicity of NK cells extracted from 3 HPV16^+^ women and 3 HPV18^+^ women was determined. As shown in Figure 3, the ratio of CFSE^+^7-AAD^+^-labeled cancer cells failed to further elevate when ten-fold ex vivo NK cells were added for coculture, indicating that infiltrated NK cells do not exert an equivalent function to eliminate transformed cells.

## 4. Discussion

The aim of this study was to prospectively study the distribution patterns of infiltrated immune cells, in particular NK cells, and HPV16- and HPV18-infected cervical surface microenvironment and to elucidate the possible high-risk HPV pathogenic mechanism from a new prospective by highlighting the functional cell discrepancies between these two types. We found that CD16^+^ NK cells were abundant in the HPV16^+^ group of women with more IFN-*γ* secretion observed. CD16 is a vital membrane protein found on NK cells that can induce HPV-VLP endocytosis followed by degranulation and cytokine secretion (such as IFN-*γ* and tumor necrosis factor (TNF)) [6, 11]. Engagement of CD16 on NK cells results in its ADCC activation [12, 13]. There results imply that the chronic inflammation-induced antibody-dependent pathway is active in cervical NK cells.

However, these increased NK cells still failed to eliminate the mature HPV virus under circumstances that Treg cells were locally restricted. It is probably due to inadequate IL-2 production which is reported to positively regulate NK cell cytotoxic function and downstream differentiation [14], as well as virus-induced KLRG-1 expression. KLRG-1 belongs to the C-lectin superfamily and is known to be an inhibitory receptor of NK cells. KLRG-1^−^ NK cells proliferate well, but they lack a mature phenotype [15]. Nevertheless, recent researches have emphasized that “NK cell education” which is part of its functional modification is mainly conducted via the ITIM/SHP1/SHP2/SHIP signaling pathway [16]. KLRG-1 is associated with the recruitment of SHIP and the KLRG/SHIP disturbance renders NK cells hyporesponsive and uneducated [17, 18]. Since effector CD8^+^ T cells also express KLRG-1, we cannot rule out a role for exhausted CD8^+^ T cells in an HPV-infected cervix [19]. We also found that HPV infection decreased IFN-*γ* transcription by immune cells, and this was consistent with other studies. It has been reported that the early gene products, E6 and E7, regulate local cytokine and chemokine expression, for instance the downregulation of IFN-*γ* and other components related to the signal transduction pathway, against virus infection [20].

Our study has some limitations: (1) cytokines were tested using quantitative real-time PCR, and this can be problematical. Some researchers collected fluid by cervicovaginal lavage in order to detect secretory proteins by ELISA. This is only feasible if vaginal diseases are first excluded; (2) the number of specimens collected was small. We enrolled women infected with either HPV16 or HPV18, but a large proportion of HPV women had a combined infection along with other low-risk types. In particular, the incidence of HPV18 itself was low in Eastern China. A future work will be focusing on evaluating other candidate cytokines (which in the present study showed a trend of change but with no statistical significance) such as IL-6, GM-CSF, CCL-5, and membrane protein DNAM-1 in HPV infection; (3) in the mixing of specimens for flow cytometry analysis, according to Hubert's study [21], the number of NK cells in cervical biopsies was too low to score, and they could only be counted in the stroma. Therefore, 4 specimens of different patients were mixed to produce a sample for flow cytometry analysis. Each group was repeated independently for four times.

One of the cervical cancer-preventive HPV vaccines—Cervarix™—is currently authorized for use in Europe and the United States. It is composed of viral components from the later period of gene expression, such as product L1 of HPV16 and HPV18 and an adjuvant, AS04. Although it has certain positive effects on the prevention of viral reinfection, its low concentration in the cervical environment is still an annoying problem and cannot be ignored. More importantly, it is far away from the curing the disease [22]. Another medicinal product used against high-risk HPV types in Canada is a quadrivalent HPV vaccine (Gardasil, Merck) which was recently proved to be 30-50% effective against lesions caused by all HPV types [23]. Over the past decade, many researchers have devoted time and effort into developing a therapeutic vaccine. Some have made gratifying results in animal models, but these vaccine applications are still confined to the laboratory. Clinical observations also suggest that antibodies in patients with persistent infection are mostly negative. Therefore, it is questionable whether humoral immune status could be used as an indicator of regression for any infection. The efficacy of therapeutic vaccines is closely related to the T cell response, Th1 cytokine secretion, local infiltration of CD4^+^ and CD8^+^ T cells, and the function of NK cells in the cervix [24]. Based on this study, we raise another question that will such therapeutic vaccines possess equal capability for killing cervical cancer cells previously infected by either HPV16 or HPV18? We believe that drugs/vaccines targeting specific HPV type will be a new promising direction for HPV treatment.

## 5. Conclusions

In summary, our data demonstrated that the number of NK cells was increased, but their cytotoxic function was abnormal in an HPV16-infected cervix. This is the first study emphasizing the unique immune profiles of the cervical microenvironment between two high-risk HPV types. The involved mechanisms may partially reveal the reason why HPV16 is the most likely to cause cervical cancer and may provide new potential strategies for its clinical management.

## Figures and Tables

**Table 1:** Quantitative real-time PCR primers used in this study.

Gene name	Primer
IFN-*γ*	F: gagtgtggagaccatcaagg
	R: cgacagttcagccatcactt

IL-2	F: gccacagaactgaaacatct
	R: gccttacctttagttccagaa

IL-6	F: agatttgagagtagtgaggaa
	R: actgtctttgagcctgtctt

GM-CSF	F: gccactacaagcagcactg
	R: tgtctgcctcctctctgga

CCL-3	F: taactcttcctcccttctcc
	R: tggacccctcaggcactca

CCL-5	F: atcctccctcttcttctcct
	R: ttcaggttcaaggactctcc

KLRG-1	F: tcaactccttttctgtgcatg
	R: catctatcaaagtctgacctt

CD56	F: atgatgggtgaagagaaccg
	R: aatgagatgtgtgtgtgtgc

CD27	F: gccttcagatgtgccctat
	R: cagtgggtagagagagtcc

DNAM-1	F: gaagtcccatctctaccagt
	R: agcttaaactctagtctttgg

*β*-Actin	F: tcatgaagtgtgacgtggacat
	R: ctcaggaggagcaatgatcttg

**Table 2:** Characteristics of HPV16 or HPV18 infections correlated to the severity cervical lesions.

Characteristics	HPV16^+^	HPV18^+^	*P* value
Average age (years)	40.0 ± 9.7	40.8 ± 9.6	0.8055
Histological diagnosis			<0.0001
Inflammation	54 (26.6)	27 (64.3)	
CIN 1	22 (10.8)	8 (19.0)	
CIN 2	29 (14.3)	3 (7.1)	
CIN 3	98 (48.3)	4 (9.5)	
Total	203	42	

CIN: cervical intraepithelial neoplasia; HPV: human papillomavirus.

## Data Availability

All data supporting the results reported in the article are generated and archived in the facilities of the Department of Laboratory Medicine, the First Affiliated Hospital of Nanjing Medical University.

## Abbreviations

HPV: Human papillomavirus
CIN: Cervical intraepithelial neoplasia
NK cell: Natural killer cell
IFN-*γ*: Interferon-*γ*
IL: Interleukin
GM-CSF: Granulocyte-macrophage colony-stimulating factor
CCL: CC-chemokine ligand
KLRG-1: Killer cell lectin-like receptor subfamily G member 1
DNAM-1: DNAX accessory molecule-1
ADCC: Antibody-dependent cellular cytotoxicity
ITIM: Immunoreceptor tyrosine-based inhibitory motif
SHP: SH2-domain-containing protein tyrosine phosphatase
SHIP: SH2-domain-containing inositol-5-phosphatase
Treg: Regulatory T cells.
